# Specific oncolytic activity of herpesvirus saimiri in pancreatic cancer cells

**DOI:** 10.1054/bjoc.2000.1346

**Published:** 2000-07-03

**Authors:** A J Stevenson, M S Giles, K T Hall, D J Goodwin, M A Calderwood, A F Markham, A Whitehouse

**Affiliations:** Molecular Medicine Unit, University of Leeds, St. James’s University Hospital, Leeds, LS9 7TF, UK

**Keywords:** pancreatic cancer, oncolytic, gene therapy, HVS

## Abstract

The potential use of oncolytic viruses in the treatment of cancer has been investigated for some time. A variety of agents have been studied, including some which appear to be selectively replication-competent in cancer cell lines. In this study, we have investigated the ability of herpesvirus saimiri to specifically lyse selected human cancer cell lines. Upon infection with a replication-competent virus carrying the EGFP reporter gene and a neomycin resistance marker, the pancreatic cancer lines MIAPACA and PANC-1 exhibited definite cytopathic effects. In contrast, the colonic carcinoma cell lines SW480 and HCT116 were phenotypically unaltered. In addition, stable cell lines could not be generated from PANC-1 infected cultures, in marked contrast to cultures of cells from other human tissues. Virus recovery assays demonstrated that all of the cell lines produced a small amount of virus post-infection, but that virus replication was minimal after 1 week in culture. In addition, treatment with acyclovir inhibited virus replication but paradoxically increased cytopathic effect. These data suggest that herpesvirus saimiri may have potential as an oncolytic agent for the treatment of pancreatic cancer. © 2000 Cancer Research Campaign


*British Journal of Cancer* (2000) **83**(3), 329–332
© 2000 Cancer Research Campaign
doi: 10.1054/ bjoc.2000.1346, available online at http://www.idealibrary.com on IDEAL®

### Specific oncolytic activity of herpesvirus saimiri in pancreatic cancer cells

**AJ Stevenson, MS Giles, KT Hall, DJ Goodwin, MA Calderwood, AF Markham and A Whitehouse**

Molecular Medicine Unit, University of Leeds, St. James's University Hospital, Leeds, LS9 7TF, UK

**Summary** The potential use of oncolytic viruses in the treatment of cancer has been investigated for some time. A variety of agents have been studied, including some which appear to be selectively replication-competent in cancer cell lines. In this study, we have investigated the ability of herpesvirus saimiri to specifically lyse selected human cancer cell lines. Upon infection with a replication-competent virus carrying the EGFP reporter gene and a neomycin resistance marker, the pancreatic cancer lines MIAPACA and PANC-1 exhibited definite cytopathic effects. In contrast, the colonic carcinoma cell lines SW480 and HCT116 were phenotypically unaltered. In addition, stable cell lines could not be generated from PANC-1 infected cultures, in marked contrast to cultures of cells from other human tissues. Virus recovery assays demonstrated that all of the cell lines produced a small amount of virus post-infection, but that virus replication was minimal after 1 week in culture. In addition, treatment with acyclovir inhibited virus replication but paradoxically increased cytopathic effect. These data suggest that herpesvirus saimiri may have potential as an oncolytic agent for the treatment of pancreatic cancer. © 2000 Cancer Research Campaign

**Keywords**: pancreatic cancer; oncolytic; gene therapy; HVS

Replication-incompetent viruses have been extensively evaluated as vectors for potential gene delivery to tumour cells, with significant preclinical efficacy having been demonstrated by direct injection of localized tumour masses in animal models (Clayman et al, 1995; Arai et al, 1997; Sanding et al, 1997). However, a major limitation is their apparent inability to effectively treat tumour metastases (Roth and Cristiano, 1997). Selectively replication-competent or oncolytic viruses hold promise for the treatment of a variety of cancers. A simple protocol would involve the virus being administered intravenously, potentially exposing many cells to infection. However, the virus would only replicate in (and selectively destroy) malignant cells. Such a procedure would be ideal for the treatment of disseminated disease, the most commonly fatal form of cancer, and would not require the physical targeting of the agent. ONYX-015, for example, is an E1B gene-deleted adenovirus which selectively replicates in and lyses cancer cells expressing mutant *p53* (Bischoff, 1996; Heise et al, 1997). This virus has recently shown antitumoural efficacy in one model of metastatic cancer (Heise et al, 1999).

The herpesviruses, primarily herpes simplex virus type 1 (HSV-1), are also under investigation as a potential source of oncolytic viruses (Rampling et al, 1998). We are currently developing gene therapy vectors based on herpesvirus saimiri (HVS). The natural host of this virus is the squirrel monkey (*Saimiri sciureus*) where it is able to persist without causing any obvious disease. However, if the virus is experimentally transmitted to other New World primates, the animals can develop a number of lymphoproliferative diseases (Fleckenstein and Desrosiers, 1982). HVS strains have been assigned to three subgroups A, B and C, depending on their transforming and pathogenic capabilities and sequence divergence of a terminal region of the viral L-DNA (Medveczky et al, 1984; 1989). In addition, viruses from subgroup C have been shown to be capable of transforming human T-cells in vitro (Beisinger et al, 1992). For reasons of safety, we have therefore evaluated the A-strain of the virus, which is incapable of transforming human T-cells. The virus has been further modified by the removal of the saimiri transforming protein (stpA), rendering it non-pathogenic and non-transforming in all systems tested to date (Desrosiers et al, 1984; 1985; 1986; Koomey et al, 1984).

It has been demonstrated that a selectable HVS genome has the ability to persist episomally in a wide variety of human cell lines for long periods of time, apparently without the production of infectious progeny viruses in many cases (Grassman and Fleckenstein, 1989; Simmer et al, 1991; Stevenson et al, 1999). In this report, we demonstrate that HVS is selectively cytopathic in pancreatic cancer cell lines, suggesting that it may have potential as a specific oncolytic agent for pancreatic cancer.

*Received 29 November 1999*
*Revised 10 March 2000*
*Accepted 26 March 2000*

*Correspondence to:* A Whitehouse

### MATERIALS AND METHODS

### Cell culture

SW480 and HCT116 cells were routinely grown in RPMI 1640 and McCoy's 5A media, respectively, supplemented with 10% fetal calf serum (FCS) and the antibiotics penicillin and streptomycin. PANC-1 and MIAPACA cells were routinely grown in Dulbecco's modified Eagle's medium (DMEM) supplemented with 10% FCS and the antibiotics penicillin and streptomycin. All tissue culture reagents were obtained from Gibco BRL (Paisley, Scotland). SW480, MIAPACA and PANC-1 cells have a mutation in the *p53* gene, in contrast to HCT116 which possess the endogenous *wt-p53* gene (Nigro et al, 1989; Redston et al, 1994).

### Virus

The marker virus HVS-GFP, containing the enhanced green fluorescent protein (EGFP) and the neomycin resistance gene (NeoR), has been described previously (Whitehouse and Stevenson, 1999).

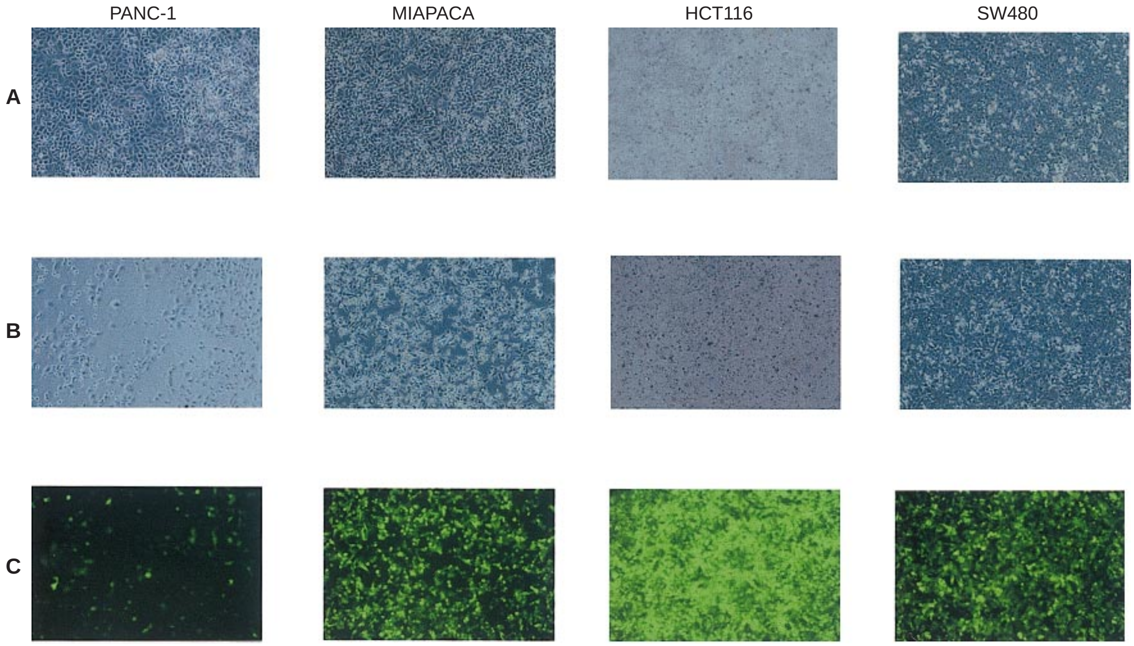

**Figure 1** Microscopic analysis of pancreatic (PANC-1 and MIAPACA) and colorectal (HCT116 and SW480) cell lines at 48 h post-infection (**A**) mock-infected cells at 48 h post-infection (**B**) 48 h post-infection with HVS-GFP at an MOI of 1 using bright field microscopy (**C**) 48 h post-infection with HVS-GFP at an MOI of 1 using FITC illumination

Briefly, it contains the EGFP gene under the control of a CMVIE promoter and the NeoR gene under the control of an SV40 promoter, cloned between unique repetitive elements of the HVS genome. The addition of these heterologous genes into the HVS genome has no detectable effect on the growth of HVS-GFP in comparison with the wild type virus.

### Infection of cells

$1 \times 10^6$ cells were infected at a multiplicity of infection (MOI) of 1 with extracellular stocks of HVS-GFP. Infections were carried out in six-welled dishes by aspirating the culture medium and adding the appropriate amount of virus in 0.5 ml of medium. The cells were then incubated at 37°C for 90 min after which a further 1.5 ml of medium was added. Cultures were examined daily for cytopathic effects (CPE).

### Drugs

Acyclovir (ACV) (Sigma, Poole, UK) was dissolved in sterile water and added to the tissue culture medium after infection with the virus. Concentrations of between 0 and 100 μM were investigated.

### Virus recovery assays

Supernatant was harvested and replaced, in infected cultures at 2, 5 and 7 days post-infection. 0.5 ml of each sample was used to infect cultures of owl-monkey kidney (OMK) cells, in which HVS is capable of forming plaques. The cultures were examined after 72 h by fluorescence microscopy for the presence of clusters of green cells (indicative of the early stages of plaque formation). This method of enumeration was possible because of the EGFP expressed by the recombinant virus. Numbers of plaques were confirmed by conventional visualization after 1 week.

### RESULTS

### Cytopathic effects in cell cultures

Examination of cells at 48 h post-infection showed significant cytopathic effects in cultures of pancreatic cancer cells (MIAPACA and PANC-1). This was in marked contrast to the colonic cancer cells (SW480 and HCT116) which exhibited no obvious CPE. Fluorescence microscopy revealed that, in all cases, the vast majority of cells had been successfully infected by the virus (i.e. exhibited a green phenotype) (Figure 1).

Extended analysis revealed that the CPE in the PANC-1 culture was progressive, leading to the total destruction of the culture at 7 days post-infection (Figure 2). Although a similar CPE was noted in the MIAPACA culture a few cells seemed to escape the effect, allowing the monolayer to recover over time. The fact that these cells were green indicated that they had been successfully infected, though not destroyed, by the virus. At no time was any CPE noted in cultures of SW480 and HCT116 cells. This result is consistent with data from other colorectal lines including HT29s, the lung carcinoma cell line A549s (data not shown) and the published results on infection of other human cell lines (Simmer et al, 1991; Stevenson et al, 1999).

The addition of ACV to infected and control cultures of MIAPACA, SW480 and HCT116 had no apparent effect, whereas in the case of infected PANC-1 cultures, increasing concentrations appeared to accelerate the process of monolayer destruction (data not shown).

### Virus recovery assay

The results of the virus recovery assay are shown in Figure 3. High numbers of green foci were produced in the OMK monolayers by all of the 2-day infected culture supernatants. However, this was

 *© 2000 Cancer Research Campaign*

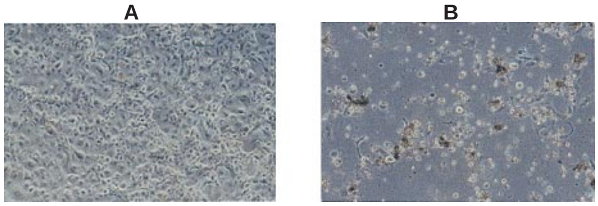

**Figure 2** Microscopic analysis of PANC-1 cells at 7 days post-infection (**A**) mock-infected cells at 7 days post-infection (**B**) 7 days post-infection with HVS-GFP at an moi of 1 using bright field microscopy

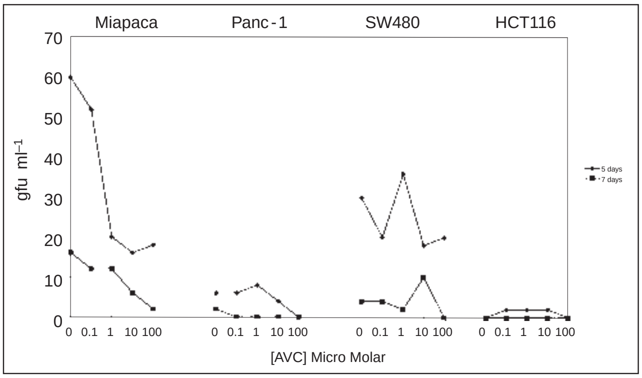

**Figure 3** Virus recovery assay. Numbers of green forming units per ml (gfu $ml^{-1}$) produced from the supernatants of infected MIAPACA, PANC-1, SW480 and HCT116 cultures were determined on days 5 and 7 post-infection in the presence of different concentrations of acyclovir. The supernatants were used to infect a permissive OMK cell line and gfu $ml^{-1}$ is an average taken from the results of two separate experiments also found with the 2-day sample from a control experiment in which medium containing virus was incubated in the absence of cells.

At 5 days post-infection, MIAPACA and SW480 cells produced the largest amounts of virus. In the absence of ACV, 60 green forming units (gfu) per ml were present in the MIAPACA culture. The presence of increasing concentrations of the drug reduced this figure to 18 gfu $ml^{-1}$. 20 gfu $ml^{-1}$ were found in the SW480 culture at the highest concentration (100 μM) of ACV, representing a less significant reduction overall. Lower numbers of gfu $ml^{-1}$ were detected in the PANC-1 culture and minimal virus was detected in the HCT116 culture at 5 days post-infection. In the case of PANC-1, the presence of 100 μM ACV resulted in no virus being recovered. As expected, virus was not recovered from the 'no-cell' control experiment after 5 days, owing to its instability at 37°C.

At 7 days post-infection, the amount of recovered virus was reduced in every case. No virus was detected in the HCT116 cultures, which remained green confirming persistence of EGFP expression. The presence of ACV prevented any virus being recovered from the PANC-1 cultures. Even in its absence only 2 gfu $ml^{-1}$ were detected. Lesser amounts of virus could still be recovered from the MIAPACA and SW480 cultures, but the presence of 100 μM ACV reduced this to very low levels in MIAPACA (2 gfu $ml^{-1}$) and to zero in SW480 cultures.

### DISCUSSION

The use of a virus as an oncolytic agent for the treatment of cancer is normally dependent on it being selectively replication-competent in the tumour cell, to prevent the destruction of healthy tissue. We are currently attempting to develop vectors for the treatment of colorectal and pancreatic cancers based on HVS, a virus which is known to be capable of infecting and persisting within many different human cancer cell lines without apparently causing CPE (Grassman and Fleckenstein, 1989; Simmer et al, 1991; Stevenson et al, 1999). In this study, we have investigated the interactions of an HVS vector expressing EGFP with cell lines representative of the above disease targets. The virus was able to efficiently infect all of the cell lines and, in three out of four instances, stably infected lines were established in which all of the cells expressed the marker protein EGFP. This finding was anticipated. However, surprisingly, the virus caused significant CPE and cell death in the pancreatic cancer cell lines (MIAPACA and PANC-1). This was so severe in the case of PANC-1 that the cell sheet was completely destroyed. Extensive destruction of the MIAPACA cells also occurred. In contrast, the colonic cancer cell lines were apparently unaffected by the infection procedure and exhibited no CPE.

In order to quantitatively assess the amount of virus, if any, produced by these infected cultures, a virus recovery assay was performed. The level of virus production after 5 days was greatest in the pancreatic cell line MIAPACA. However, the destruction of the PANC-1 monolayer at earlier times post-infection suggested that significant virus production may well have occurred prior to the 5-day sample. Unfortunately, as previously discussed, virus production at early stages could not be distinguished from input virus. According to control experiments, input virus was not totally inactivated until 5 days post-infection. Considering the relatively low levels of virus that were produced in any of the cultures it seems possible that the CPE observed in the pancreatic cell lines is not directly linked to the levels of virus production and may instead be caused by the expression of a virus gene product to which the cells are particularly sensitive. The expression of herpes simplex virus type 1 immediate early genes, for example, is known to cause CPE in a variety of human cells (Johnson et al 1994).

One of the advantages of using a herpesvirus-based vector in a clinical setting is that replication of the virus can be controlled by using the drug ACV (Elion, 1980). Our studies have shown that ACV has activity against HVS and could therefore be used as a safeguard in any future clinical investigation. In addition, ACV appeared to accelerate the destruction of the PANC-1 culture. This observation may be due to its activation in cells which were expressing the HVS thymidine kinase gene product, thereby contributing to the death of the cell. A similar phenomenon is widely exploited by 'suicide' gene therapy strategies, involving the use of the HSV-TK gene product (Culver et al, 1992).

In summary, we have shown that HVS appears to be selectively cytopathic in pancreatic tumour cells. This unexpected result suggests its possible application as an oncolytic agent for the treatment of pancreatic cancer. If the virus, in its present replication-competent form, was inoculated directly into a tumour it would be unlikely to spread far from the injection site owing to the low levels of virus production from infected human cells. We also note that accidental inoculations into human subjects of the most potently transforming strain of HVS have not resulted in sero-conversion of the individuals involved or any recognizable disease (Fleckenstein and Desrosiers, 1982). The strain of virus used in these studies has already been attenuated, being incapable of transforming any known cell type examined to date or causing pathology in any animal model so far tested. HVS therefore merits further evaluation with a view to possible exploitation of these

*© 2000 Cancer Research Campaign* 

serendipitous findings for the treatment of this most intractable malignancy.

### ACKNOWLEDGEMENTS

Work in the authors' laboratory is supported by the Medical Research Council (MRC), Wellcome Trust, Candlelighters Trust and Yorkshire Cancer Research. A Whitehouse and D Goodwin are the recipients of an MRC Fellowship and MRC Studentship, respectively. M Giles is a Wellcome Trust Clinical Research Training Fellow.

### REFERENCES

Arai H, Gordon D, Nabel EG and Nabel GJ (1997) Gene transfer of the Fas ligand induces tumour regression in vivo. *Proc Natl Acad Sci USA* **94**: 13862–13867

Biesinger B, Muller-Fleckenstein I, Simmer B, Lang G, Wittmann S, Platzer E, Desrosiers RC and Fleckenstein B (1992) Stable growth transformation of human T lymphocytes by herpesvirus saimiri. *Proc Natl Acad Sci USA* **89**: 3116–3119

Bischoff JR, Kirn DH, Williams A, Horn S, Muna M, Ng L, Nye JA, Sampson-Johannes A, Fattaey A and McCorrmick F (1996) An adenovirus mutant that replicates selectively in p53-deficient human tumour cells. *Science* **274**: 373–376

Clayman GL, el-Nagger AK, Roth JA, Goepfert H, Taylor DL and Liu TJ (1995) In vivo molecular therapy with p53 adenovirus for microscopic residual head and neck squamous carcinoma. *Cancer Res* **55**: 1–6

Culver KW, Ram Z, Walbridge S, Ishii H, Oldfield EH and Blaese RM (1992). In vivo gene transfer with retroviral vector-producer cells for the treatment of experimental brain tumours. *Science* **256**: 1550–1552

Desrosiers RC, Burghoff R, Bakker A and Kamine J (1984). Construction of replication-competent herpesvirus saimiri deletion mutants. *J Virol* **49**: 343–348

Desrosiers RC, Bakker A, Kamine J, Falk L, Hunt R and King N (1985) A region of the herpesvirus saimiri genome required for oncogenicity. *Science* **228**: 184–187

Desrosiers RC, Silva D, Waldron L and Letvin N (1986) Non-oncogenic deletion mutants of herpesvirus saimiri are defective for in vitro immortalization. *J Virol* **57**: 701–705

Elion GB (1980) The chemotherapeutic exploitation of virus-specified enzymes. *Adv Enzyme Regul* **18**: 53–66

Fleckenstein B and Desrosiers RC (1982) Herpesvirus saimiri and herpesvirus ateles. In: *The Herpesviruses, Vol 1*, Roizman B (ed), pp. 253–332. Plenum Press: New York

Grassmann R and Fleckenstein B (1989) Selectable recombinant herpesvirus saimiri is capable of persisting in a human T-cell line. *J Virol* **63**: 1818–1821

Heise CC, Sampson JA, Williams A, McCormick F, Von HD and Kirn DH (1997) ONYX-015, an E1B gene-attenuated adenovirus, causes tumour-specific cytolysis and antitumoural efficacy that can be augmented by standard chemotherapeutic agents. *Nat Med* **3**: 639–645

Heise CC, Williams A, Olesch J and Kirn DH (1999) Efficacy of a replication-competent adenovirus (ONYX-015) following intratumoral injection: intratumoral spread and distribution effects. *Cancer Gene Ther* **6**: 499–504

Johnson PA, Wang MJ and Friedmann T (1994) Improved cell survival by the reduction of immediate-early gene expression in replication-defective mutants of herpes simplex virus type 1 but not by mutation of the virion host shutoff function. *J Virol* **68**: 6347–6362

Koomey J, Mulder C, Burghoff R, Fleckenstein B and Desrosiers RC (1984) Deletion of DNA sequences in a non-oncogenic variant of herpesvirus saimiri. *J Virol* **50**: 662–665

Medveczky P, Szomolanyi E, Desrosiers RC and Mulder C (1984) Classification of herpesvirus saimiri into 3 groups based on extreme variations in a DNA region required for oncogenicity. *J Virol* **52**: 938–944

Medveczky MM, Szomolanyi E, Hesselton R, DeGrand D, Geck P and Medveczky PG (1989) Herpesvirus saimiri strains from three DNA subgroups have different oncogenic potentials in New Zealand white rabbits. *J Virol* **63**: 3601–3611

Nigro JM, Baker SJ, Preisinger AC, Jessup JM, Hostetter R, Cleary K, Bigner SH, Davidson N, Baylin S, Devilee P, Clover T, Collins FS, Weston A, Modali R, Harris CC and Vogelstein B (1989). Mutations in the p53 gene occur in diverse human tumour types. *Nature* **342**: 705–708

Rampling R, Cruickshank G, Maclean A and Brown M (1998) Therapeutic replication-competent herpes virus. *Nat Med* **4**: 133

Redston MS, Caldas C, Seymour AB, Hruban RH, da Costa L, Yeo CJ and Kern SE (1994) p53 mutations in pancreatic carcinoma and evidence of common involvement of homocopolymer tracts in DNA microdeletions. *Cancer Res* **54**: 3025–3033

Roth J and Cristiano RJ (1997) Gene therapy for cancer: what have we done and where are we going? *J Natl Cancer Inst* **89**: 21–39

Sandig V, Brand K, Herwig S, Lukas J, Bartek J and Strauss M (1997) Adenovirally transferred p16INK4/CDKN2 and p53 genes cooperate to induce apoptotic tumour cell death. *Nat Med* **3**: 313–319

Simmer B, Alt M, Buckreus I, Berthold S, Fleckenstein B, Platzer E and Grassmann R (1991) Persistence of selectable herpesvirus saimiri in various human haemopoietic and epithelial cell lines. *J Gen Virol* **72**: 1953–1958

Stevenson AJ, Cooper M, Griffiths JC, Gibson PC, Whitehouse A, Jones EF, Markham AF, Kinsey SE and Meredith DM (1999) Assessment of herpesvirus saimiri as a potential human gene therapy vector. *J Med Virol* **57**: 269–277

Whitehouse A and Stevenson AJ (1999) Gene regulation in Herpesvirus saimiri and its implication for the development of a novel gene therapy vector. *Gene Therapy and Molecular Biology* **3**: 35–44

 *© 2000 Cancer Research Campaign*
